# Editorial: Modelling and sensing platform for cancer and tumour microenvironment

**DOI:** 10.3389/fbioe.2024.1432352

**Published:** 2024-06-04

**Authors:** Yuji Nashimoto, Yusuke Kanno, Hidenori Ito, Mustafa ŞEN

**Affiliations:** ^1^ Institute of Biomaterials and Bioengineering (IBB), Tokyo Medical and Dental University (TMDU), Tokyo, Japan; ^2^ Institute of Innovative Research, Tokyo Institute of Technology, Yokohama, Kanagawa, Japan; ^3^ RIKEN BioResource Research Center (BRC), Tsukuba, Ibaraki, Japan; ^4^ Department of Biomedical Engineering, Izmir Kâtip Çelebi University, İzmir, Türkiye

**Keywords:** tumor model, tumor microenevironment, cancer stem cell (CSC), microphysiological system (MPS), organ-on-a-chip (OOC), microfluidic device, biosensors, tissue engineering

There is considerable interest in identifying alternatives to current animal models because of their cost, time-consuming nature, ethical concerns, and notably, the frequent inability of animal data to accurately predict human responses. Recent advances in bioengineering and stem cell culture techniques have introduced innovative tools for simulating human clinical trials. Organoid technology, which involves culturing normal or cancerous stem cells, facilitates the development of self-organising structures that emulate critical aspects of architecture, cell types, and functionality. Microphysiological systems (MPSs), also known as organs-on-chips (OoCs), represent another highly promising avenue for cancer modelling. These systems feature perfusable microchannels inhabited by living cells that mimic the organ-level physiology and pathophysiology by replicating the tumour microenvironment (TME) during cancer development. The development of sensitive detection systems capable of providing quantitative outputs is essential for the practical application of novel cancer models for drug screening. Optical, electrical, and electrochemical sensors have been developed and integrated into the new cancer models. This study aims to highlight the latest developments in cancer modelling and biosensing and their practical applications, including predicting human responses, understanding the intricacies of cancer biology, quantitatively measuring biological activities, and facilitating the practical application of these models in drug screening.

Microengineering techniques have been utilised to model physiological and pathophysiological microenvironments in this field. Recent advances in shape-morphing materials allow for the more precise modulation of architecture, flexibility, functionality, and other properties. Mirzababaei et al. overviewed manufacturing methods, structures, and biological applications of 3D assemblies composed of the shape-morphing and biomaterials. Focusing on lithography and bioprinting, these manufacturing methods are systematically organised in relation to the assembly scale, fabrication throughput, and materials. Although 3D assemblies can be manufactured directly by bioprinting, they can also be transformed from 2D substrates, and the transformational structures can be classified into three types: rolling, gripping, and folding. The structural transformation benefits from the unique properties of shape-deforming materials induced by stimuli such as temperature and pH. In addition, 3D assemblies can be dynamically transformed by specific stimuli, such as 4D assemblies. These 3D and 4D assemblies have been widely applied in minimally invasive surgery for anastomosis and biopsy as well as in biomodels, implants, and biosensors. The need for further exploration of gas permeability and interactions with the immune system was emphasised to further improve the reproducibility and affinity of the assemblies for biological organisms. We believe that the above comprehensive discussion is crucial for the development of the modelling and sensing of cancer and tumour environments.

Organ decellularization and recellularization are emerging approaches for creating bioengineered tissues to recapitulate the functions *in vitro*. Mizoguchi et al. developed an *ex vivo* 3D lung cancer model using decellularization and recellularization methods to simulate human lung tissue complexities. Initially, rat lungs were decellularised to create scaffolds, and subsequently reseeded with epithelial, endothelial, and adipose-derived stem cells to generate bioengineered rat lungs. Four human lung cancer cell lines (A549, PC-9, H1299, and PC-6) were introduced into bioengineered rat lungs to establish a 3D lung cancer model. Histopathological examination revealed varied nodule morphologies, indicating the diverse characteristics of the seeded lung cancer cells, which were influenced by their microenvironments. Notably, A549 cells cultured on recellularised lungs exhibited significantly elevated expression of mucin 1 (MUC-1), a protein linked to lung carcinoma, compared with both 2D culture and decellularised lungs, confirming the ability of the model to reproduce *in vivo* features. RNA sequencing further confirmed MUC-1 mRNA upregulation in 3D cultures, irrespective of recellularisation. Additionally, drug testing with epidermal growth factor receptor-thyroxin kinase inhibitors exhibited selective suppression of cell proliferation, highlighting the effectiveness of this model for testing anticancer drugs. Despite its reliance on rat-derived cells and established cell lines, this model provides a controlled setting for investigating tumour dynamics and treatment responses, similar to the human lung microenvironment.

MPS or OoC technologies hold great promise for recapitulating cell-cell interactions in the TME. Jiang et al. investigated the conversion of stromal fibroblasts into cancer-associated fibroblasts (CAFs) in their prostate-cancer-on-chip (PCoC) model, in which human prostate cancer and stromal fibroblast cells were co-cultivated in two channels separated by a porous membrane. Among the tumour cell lines investigated, including C4-2, a human prostate cancer cell line, and primary tumour cells derived from a patient, the upregulation of CAF markers, such as alpha-smooth muscle actin (αSMA) and collagen type I alpha 1 (COL1A1) was observed. In contrast, the non-cancerous epithelial cells (immortalised primary human prostate basal epithelial cells (PrECs)) did not induce the upregulation. They further investigated tumour invasion into the stroma, an early step in the metastatic cascade. Both tumour cells and CAFs were observed to cross over into their neighbouring channels, which became more aggressive when in direct contact. The presence of TGF-β detected in the culture medium was postulated as a potential mediator of the observed CAF transformation and subsequent invasive activity.

Identifying subpopulations within an intact tumour is crucial for developing advanced screening platforms to evaluate variations in treatment responses among these subpopulations. Nashimoto et al. employed scanning electrochemical microscopy (SECM) to assess intratumor heterogeneity and plasticity in patient-derived tumour organoids. SECM, recognised as a noninvasive analytical instrument, facilitates the measurement of oxygen consumption rates (OCR) in cellular aggregates, including embryos and tumour spheroids. Utilising patient-derived cancer organoids, which exhibit diverse growth potentials established through cancer tissue-originated spheroid methodology, they modelled tumours with heterogeneous subpopulations. The SECM provided detailed analyses of OCRs in individual organoids as small as 100 µm in diameter, uncovering heterogeneity within the subpopulations that was not evident in traditional colorectal cancer cell lines. Moreover, their analysis of oxygen metabolism in pre-isolated subpopulations with slow growth potential demonstrated that variations in the OCR may indicate differences in the organoid growth rate. Electrochemical measurements, including this technology, are expected to become vital tools for differentiating tumour subpopulations and developing innovative drug-screening platforms.

In addition to emerging models, traditional animal models are still indispensable tools because new *in vitro* models cannot fully recapitulate whole-body reactions. Hori et al. described an important mechanistic understanding of constitutive active/androstane receptor (CAR)-dependent hepatocellular carcinoma (HCC) promotion in a genetically modified mouse model. Growth arrest and DNA-damage inducible 45 beta (Gadd45β) is an interactor with CAR protein and serves as a scaffold protein for regulating the activity of mitogen-activated protein kinase (MAPK). They studied Gadd45β null mice treated with N-diethylnitrosamine (DEN) and phenobarbital (PB) to investigate the role of Gadd45β in PB-mediated HCC development. Subsequently, they demonstrated that PB-mediated HCC was only induced in the wild-type mice but not in Gadd45β null mice, suggesting that Gadd45β was essential for PB-mediated HCC tumorigenesis. Moreover, they performed a microarray analysis of mRNA derived from the mouse livers and identified two genes, tgfbr2 and irisin/Fndc5, which were upregulated in the wild-type mice; however, no significant increase was observed in Gadd45β null mice. In fact, the expression of Tgfbr2 and irisin/Fndc5 enhanced the growth of the human HCC cell line HepG2. These findings suggest that Gadd45β plays a critical role in HCC development by regulating the irisin/Fndc5 and Tgfbr2 genes.

Overall, this study demonstrated diverse cutting-edge methodologies, including shape-morphing materials (Mirzababaei et al.), decellularization (Mizoguchi et al.), MPS (Jiang et al.), and biosensing (Nashimoto et al.), while also highlighting the contributions of traditional animal models (Hori et al.). These innovative engineering and sensing tools will catalyse further developments in tumour biology, providing a more profound and comprehensive understanding of the field. We thank all authors and reviewers who have enriched this Research Topic as guest editors. We anticipate that this Research Topic will significantly benefit future studies in this field.

